# Development and validation of the reported outcome scale for patients with lumbar disc herniation

**DOI:** 10.1080/07853890.2025.2524088

**Published:** 2025-06-28

**Authors:** Chao Xu, Feng Huang, Shunxing Wang, Zhijun Tan, Haiyue Zhang, Zhe Yang, Ying Liang, Zhou Lu, Lei Shang

**Affiliations:** a Department of Health Statistics, Faculty of Preventive Medicine, Fourth Military Medical University, Xi'an, China; b Department of Knee Joint Surgery, Honghui Hospital, Xi'an Jiaotong University, Xi'an, China; c Airforce Medical Center, Fourth Military Medical University, Haidian district, Beijing, China; d Xi'an Medical University, Xi'an, China; e Ministry of Education Key Lab of Hazard Assessment and Control in Special Operational Environment, Fourth Military Medical University, Xi'an, China; f Airforce Health Service Training Base, Fourth Military Medical University, Xi'an, China

**Keywords:** Lumbar disc herniation, patient reported outcome, scale preparation, reliability, validity

## Abstract

**Objective:**

The objective of this study was to develop a culturally relevant and clinically robust Patient-Reported Outcome (PRO) scale for patients with lumbar disc herniation (LDH), supporting standardized disease monitoring and quality of life assessment.

**Methods:**

An initial 54-item pool was generated through literature review, patient interviews, and expert consultation. In the first round, 163 LDH patients were surveyed, and items were screened using exploratory factor analysis, critical ratio analysis, Cronbach's α, Pearson correlation, and discrete trend analysis. This yielded a 29-item trial scale. In the second round, 350 patients completed the scale for further refinement. In the third round, 539 patients were assessed for test–retest reliability and internal consistency. Validity was examined through content and criterion-related validity analyses.

**Results:**

The final PRO scale comprised 29 items across five dimensions: Physical Symptoms (5), Physical Dysfunction (11), Psychological emotion (5), Social Adaptation (4), and Therapeutic Effect (4), explaining 69.07% of total variance. The overall Cronbach's α coefficient was 0.95, with subscale values between 0.81 and 0.94. Test–retest reliability was 0.85. Content validity was strong, with content validity ratios from 0.60 to 1.00 and content validity indices from 0.90 to 1.00. Criterion-related validity was supported by correlations of 0.74 with the Oswestry Disability Index and 0.62 with the Self-Rating Anxiety Scale.

**Conclusion:**

The developed PRO scale is comprehensive, reliable, and valid for assessing the physical, psychological, and social impacts of LDH. It is well-suited for clinical use and quality of life evaluation in LDH patients.

## Introduction

Lumbar disc herniation (LDH) was identified as a common degenerative condition affecting the lumbar intervertebral disc [1]. The primary clinical manifestations of LDH included lumbar pain, which could radiate to one or both lower limbs and, in severe cases, result in numbness, muscle weakness, muscle atrophy, and other neurological deficits in the lower extremities [2]. Epidemiological studies reported a global incidence rate of approximately 2%–3% [3], with a higher prevalence of 7.6% observed in China [4]. Among individuals aged 30–50 years, the condition was found to be more prevalent in men than in women [5,6].

The pain and physical dysfunction associated with LDH were shown to significantly affect patients' physical well-being (manifested as pain and functional impairment), psychological well-being (including anxiety and depression), and social well-being (impacting travel, housework, and occupational activities) [7]. Previous research indicated that patients with LDH frequently experienced elevated levels of anxiety and depression, with reported prevalence rates of depression ranging from 35% to 41% and anxiety around 35% [8,9].

Within the framework of the current biopsychosocial medical model, the evaluation of disease and health status has expanded beyond biological indicators to include both medical and patient-reported outcomes (PROs), as well as quality-of-life assessments. In February 2006, the United States Food and Drug Administration (FDA) issued draft guidance regarding the use of PROs in the development and evaluation of new therapeutic agents. PROs were defined as direct reports from patients concerning various aspects of their health status, without interpretation by clinicians or others [10].

PROs have gained increasing importance in multiple areas of healthcare, including drug regulation, therapeutic evaluation, clinical trials, health technology assessment, and the appraisal of medical service quality [9,11]. Current evaluations of LDH have employed both qualitative and quantitative methods; however, existing assessment tools often fail to capture the full spectrum of patient well-being. Qualitative assessments have traditionally relied on patient interviews and physical examinations, which tend to yield subjective interpretations and may overlook the psychological and social dimensions of the disease. Consequently, these methods have predominantly focused on physical symptoms such as pain and dysfunction, potentially neglecting critical aspects of the patient experience.

Conversely, quantitative evaluations employed scales such as the Oswestry Disability Index (ODI) [12,13], the Japanese Orthopaedic Association scores (JOA) [14,15], and the Roland Morris Disability Questionnaire (RMDQ) [16,17]. While these instruments provided valuable insights into pain and functional impairment, they primarily assessed physical aspects and inadequately addressed psychological factors, treatment effectiveness, and social adaptability [18–20]. In addition, generic quality-of-life scales such as the EQ-5D and the SF-36, although applicable across various disease populations, were limited in capturing specific symptoms and treatment satisfaction pertinent to patients with LDH [21,22]. These limitations highlighted the need for a novel PRO scale specifically designed for LDH, one that incorporated the multifaceted nature of the disease and aligned with established guidelines for PRO development.

The primary objective of this study was to develop a PRO scale tailored specifically for patients with LDH and to evaluate its psychometric properties, including reliability and validity. Initially, the study aimed to construct a comprehensive assessment tool that evaluated the health status of LDH patients across physical, psychological, and social domains, with particular attention to the cultural characteristics of the Chinese population. This scale was intended to address the deficiencies of existing instruments by offering a more holistic and culturally appropriate assessment framework. Subsequently, the scale was subjected to rigorous psychometric testing to ensure its robustness in measuring relevant outcomes. Ultimately, the goal was to provide healthcare professionals with a reliable instrument for monitoring disease progression, evaluating treatment efficacy, and informing clinical decision-making, thereby improving the management and quality of life of patients with LDH.

## Methods

### Ethical approval and informed consent

This study was approved by the Medical Ethics Committee of Air Force Medical University (Approval No. XJ83313927) on 25 August 2021. The research was conducted in accordance with the ethical principles outlined in the Declaration of Helsinki. Written informed consent was obtained from all individual participants prior to their participation in the study.

### Development of conceptual model

To develop a conceptual model for PRO in LDH, a systematic process grounded in qualitative research methodology was employed. The research objectives were initially defined to explore the specific attributes of PRO within the context of LDH, and these objectives guided the entire interview process. Semi-structured interview guidelines were developed based on these objectives, incorporating open-ended questions designed to elicit participants' perceptions and experiences regarding health-related quality of life issues associated with LDH.

A theoretical sampling strategy was adopted to ensure representativeness, involving the intentional selection of a diverse sample that included six experts in orthopedics, psychology, rehabilitation medicine, and integrative medicine, as well as 20 patients diagnosed with LDH. This heterogeneous group was selected to capture a broad range of insights and experiences relevant to the research aims.

Interviews were conducted by trained interviewers with expertise in effective communication and group facilitation. This ensured that participants could openly share their experiences in a manner that safeguarded their privacy and emotional well-being. All interviews were audio-recorded and transcribed verbatim. The data were analyzed using qualitative analytical techniques to identify and construct theoretical concepts related to LDH symptoms and their impacts.

Based on the interview data, an initial conceptual model was developed that reflected the lived experiences of participants, identifying key influencing factors and their interrelationships relevant to PRO in LDH. The accuracy and comprehensiveness of the conceptual model were subsequently validated through follow-up interviews with participants and expert consultations. Feedback from these sessions informed iterative modifications aimed at enhancing the model's relevance and applicability to individuals with LDH.

Following multiple rounds of revision and validation, a final conceptual model was established. This model serves as the foundation for the development of PRO measurement tools specific to LDH. The iterative development process ensured that the conceptual model accurately represented the perspectives of participants and provided a comprehensive framework for future research and instrument development in the context of PRO for LDH.

### Creation of item pool and scale

To develop a comprehensive item pool and scale for assessing PRO in individuals with LDH, a synthesis of functional, psychological, and quality-of-life dimensions specific to LDH patients was conducted. Established measurement instruments were reviewed, and cultural nuances relevant to the Chinese context were taken into account. As a result, a preliminary draft of items was compiled for further evaluation.

The item pool underwent a feedback process involving 20 patients with LDH and six multidisciplinary experts in orthopedics, psychology, rehabilitation medicine, integrative medicine, and orthopaedics. Both experts and patients independently evaluated the items, providing valuable insights that contributed to the refinement of the item pool. This refinement process involved eliminating redundant items, enhancing clarity and representativeness, and modifying specific items to better capture the lived experiences of LDH patients.

To ensure cultural appropriateness, several adaptations were integrated into the item development process. Initially, item refinement was guided by expert consultations and patient interviews, with careful attention paid to language nuances and culturally specific expressions related to pain perception, psychological distress, and social functioning. Given the cultural emphasis on family support within Chinese society, items reflecting the impact of LDH on family relationships were incorporated. Moreover, treatment expectations were considered, recognizing the widespread use of traditional Chinese medicine and non-surgical therapies. Consequently, the scale included items addressing patient adherence to conservative treatments and their perceived effectiveness.

Following a thorough review and refinement process, the draft scale was finalized. A five-point Likert scale was adopted to measure item responses. This format was selected for its balance between reliability and ease of use, offering sufficient response variation while avoiding excessive complexity. Research has demonstrated that a five-point scale yields superior performance compared to shorter formats, such as three-point scales [23]. Although longer scales, such as seven-point Likert scales, may offer increased granularity, no substantial psychometric benefits have been observed when using scales with more than six options [24].

The scale employed five response levels across different items. For instance, frequency was assessed with options ranging from 'Never' to 'Occasionally', 'Sometimes', 'Often', and 'Always'. Pain levels were evaluated using descriptive anchors such as 'No pain' and 'Nothing painful can be done'. Similar Likert-style formats were applied to dimensions including difficulty, impact, improvement, and satisfaction. Each response option was assigned a numerical score from 1 to 5, allowing for nuanced evaluation of each domain. The user-friendly design of the five-point scale enabled efficient data collection and patient participation in clinical environments, thereby supporting its practical applicability.

### First-round survey using the draft scale

From February to March 2022, a convenience sampling method was employed to recruit 200 patients diagnosed with LDH from the outpatient department of a major comprehensive hospital in Beijing. The sample size was determined in accordance with established guidelines suggesting a minimum of 5–10 participants per item for the development of psychometric instruments [25]. Given that the initial conceptual framework for the PRO scale comprised 40 items, a sample of 200 participants was deemed sufficient to ensure robust statistical analysis and enhance the generalizability of the results. The preliminary version of the scale was used in this first round of data collection.

Patients were eligible for inclusion if they were over 18 years of age, had a confirmed diagnosis of LDH by the hospital, provided informed consent, and demonstrated sufficient cognitive capacity to complete the questionnaire. Exclusion criteria included the presence of other orthopedic or chronic conditions that could confound quality of life and daily functioning, pregnancy, age over 75 years, and significant communication barriers.

The diagnostic criteria for LDH were based on a comprehensive evaluation of clinical symptoms, physical examination findings, and imaging results [3]. A diagnosis was confirmed when at least three of the following five clinical criteria were met in conjunction with imaging evidence of disc herniation and nerve compression: (1) radiating lower limb pain consistent with nerve distribution; (2) paresthesia with reduced cutaneous sensation in the affected dermatome; (3) positive straight-leg raise test or femoral nerve stretch test on the unaffected side; (4) diminished tendon reflexes on the affected side; and (5) reduced muscle strength.

Two trained triage nurses served as data collectors. Prior to initiating the survey, they received standardized training to ensure consistent implementation. The training included an overview of the study objectives, detailed inclusion and exclusion criteria, and instructions on accurately administering the questionnaire. Eligible patients were interviewed in person using an electronic questionnaire system. Upon completion, the survey was reviewed by the investigator to ensure data integrity, and patient responses were subsequently anonymized and coded.

The questionnaire consisted of two main components: (1) demographic data including age, gender, geographical location, and education level, and (2) the initial version of the LDH-specific PRO scale. This draft scale contained 34 items across nine domains: Physical symptom, physical dysfunction, emotional well-being (anxiety and depression), social engagement, family support, work-related impact, treatment adherence, and perceived treatment efficacy.

A rigorous and systematic approach was employed to refine item content based on classical test theory. The process included the following steps:

1. Data Cleaning: Questionnaires with abnormal completion times or extreme response patterns (ceiling or floor effects) were excluded.
2. Item Screening Methods:
  Discrete Trend Method: Evaluated item sensitivity using standard deviation or coefficient of variation; items with SD ≤ 0.85 were considered insufficiently variable and removed.
  Correlation Coefficient Method: Assessed item independence and representativeness using Pearson's correlation; items with |*r*| ≤ 0.4 or high redundancy were excluded.
  Critical Ratio Method: Assessed item discrimination by comparing high- and low-scoring groups; items with non-significant differences (*p* > 0.05) or poor discrimination (*t* ≤ 3) were discarded.
  Cronbach's α Coefficient Method: Items that reduced internal consistency were removed if their deletion significantly improved the overall Cronbach's α.
  Exploratory Factor Analysis: Items with low factor loadings (≤0.4) or overlapping conceptual content were excluded.
3. Expert Review: After statistical screening, a multidisciplinary expert panel reviewed the retained items, discussing necessary revisions and adjustments. This step ensured content relevance, clarity, and comprehensiveness, culminating in the development of a refined preliminary version of the scale.

This rigorous multi-step process ensured that the item pool was optimized for content validity, internal consistency, and psychometric robustness, laying a strong foundation for subsequent validation phases.

### Second-round survey using the trial scale

The second round of data collection was conducted from August to October 2022 at a major general hospital in Beijing. During this phase, the trial version of the PRO scale was administered to both outpatient and inpatient populations. A total of 371 additional patients diagnosed with LDH were recruited using the same convenience sampling method employed in the first round. The expansion of the sample size in this phase was intended to validate the preliminary findings and enable a more comprehensive assessment of the scale's psychometric properties across a more heterogeneous patient population. The same inclusion and exclusion criteria as the first round were strictly adhered to.

A team comprising five outpatient triage nurses and ward nurses was designated to serve as investigators. These individuals received prior training and conducted face-to-face interviews with patients who met the eligibility criteria. Upon survey completion, each questionnaire was reviewed jointly by the investigator and the patient to ensure completeness and accuracy.

The survey instrument consisted of two sections: (1) demographic information, including variables such as age, gender, place of residence, and educational background, and (2) PRO as assessed by the trial version of the LDH-specific PRO scale.

Consistent with the first-round methodology, item screening in the second round followed the principles of classical test theory. In the first round, exploratory factor analysis (EFA) was applied to identify the latent structure underlying the initial scale items. EFA is a fundamental technique in psychometric evaluation, useful for revealing underlying constructs and assessing the interrelationships among observed variables.

Building upon the results of the initial analysis, the second-round data underwent an enhanced psychometric assessment that integrated EFA with parallel analysis to rigorously determine the dimensionality of the formal scale. Parallel analysis is a robust method for validating factor retention decisions by comparing the eigenvalues obtained from actual data with those derived from randomly generated datasets. This technique reduces the likelihood of over-extraction of factors, which can otherwise lead to spurious model complexity and misinterpretation.

The parallel analysis procedure involved generating two sets of eigenvalue distributions: one from the empirical dataset and another representing the average eigenvalues from a large number of random matrices. By visually comparing the scree plots of actual and random data, the intersection point was identified to determine the maximum number of factors that accounted for non-random variance. This approach provides a statistically sound criterion for factor retention, ensuring that each retained factor represents meaningful structure rather than random noise.

Additionally, the cumulative variance contribution rate was taken into account, with an emphasis on achieving a sufficiently high proportion of explained variance. This enhances the interpretability and reliability of the final scale by ensuring that the retained factors collectively account for a significant portion of the total variance observed in patient responses.

Through this combined analytical strategy, the structure of the formal LDH-specific PRO scale was refined and validated, laying a solid empirical foundation for its subsequent application in clinical and research settings.

### Verification of the reliability and validity of the formal scale

From October 2022 to January 2023, we conducted a survey of patients diagnosed with LDH who were receiving outpatient or inpatient care at comprehensive hospitals in Beijing and Ya'an. A convenience sampling method was employed, and the sample size was determined according to the standard guideline recommending a minimum of 10 participants per item in the formal scale. To evaluate test–retest reliability, a subset of 35 patients undergoing conservative treatment was randomly selected and reassessed two weeks after the initial survey. Additionally, 70 patients were randomly selected to complete the ODI and the Self-Rating Anxiety Scale (SAS) alongside our scale to assess convergent validity.

The survey was administered by two trained nurses who were instructed on the study's objectives, eligibility criteria, and standardized procedures for questionnaire administration. Eligible participants were interviewed face-to-face using electronic questionnaires. Upon completion, investigators reviewed the responses to ensure accuracy and completeness. The data were then automatically coded for subsequent statistical analysis.

#### Reliability analysis

Reliability refers to the degree of consistency, stability, and reproducibility of a measurement instrument, typically evaluated through internal consistency and test–retest reliability [26]. Higher reliability coefficients indicate greater measurement stability and precision.

Internal Consistency: Cronbach's alpha coefficient was used to assess the internal consistency of the scale. A Cronbach's alpha value of ≥0.80 is generally accepted as indicative of good internal consistency [27], suggesting that items within each domain consistently measure the same underlying construct.

Test–Retest Reliability: To evaluate temporal stability, the same group of participants (*n* = 35) completed the scale twice, with a two-week interval between assessments. The intraclass correlation coefficient (ICC) was calculated between the two sets of scores. A coefficient >0.75 was interpreted as demonstrating good test–retest reliability [28], indicating the scale's stability over time.

#### Validity analysis

Validity pertains to the degree to which the instrument accurately captures the intended construct. In this study, we examined content validity and criterion-related validity, two commonly employed metrics for scale validation.

Content validity: A multidisciplinary panel of 10 experts from orthopedics, rehabilitation medicine, and psychology evaluated each item of the scale for relevance and representativeness. The Content Validity Ratio (CVR) and Content Validity Index (CVI) were calculated based on expert ratings. A CVR value ≥0.60 was considered indicative of satisfactory content validity [29], ensuring that each item appropriately reflected the domain it was designed to measure.

Criterion-related validity: To assess criterion-related validity, the scale was compared with two established instruments—the ODI and SAS—by analyzing the correlation between their respective scores. Stronger correlation coefficients suggested better criterion validity [30], demonstrating that the new scale reliably aligns with established measures of functional disability and psychological distress.

#### Statistical analysis

Parallel analysis for factor structure determination was conducted using R software version 3.3.2, while all other statistical analyses, including reliability and validity assessments, were performed using SPSS version 23.0.

## Result

### Conceptual model of PRO

The conceptual model of PRO for patients with LDH offers a comprehensive, multidimensional framework for assessing the patient experience across key domains.

Physiological domain: This domain focuses on evaluating physical symptoms and functional impairments, which are critical indicators of disease severity.

Psychological emotion domain: This domain assesses emotional responses, including anxiety and depression, which are frequently associated with chronic pain conditions such as LDH and can significantly influence treatment adherence and recovery trajectories.

Social domain: The social dimension examines the extent to which LDH disrupts patients' participation in social roles, family responsibilities, and occupational activities. Such disruptions not only affect overall quality of life but may also contribute to psychological distress and diminished functional recovery.

Treatment-related domain: This domain evaluates perceived treatment outcomes, including symptom relief, improvements in daily functioning, adherence to treatment protocols, and satisfaction with care.

Together, these four domains constitute an integrated framework that facilitates a nuanced understanding of LDH's impact on patients. The model provides a theoretical basis for developing patient-centered measurement tools and guiding clinical decision-making (Table 1).

**Table 1.** Conceptual model of outcomes reported by patients with LDH.

	Fields	Dimensions
Outcomes reported by patients with LDH	Physiological	Physical symptoms, Functional limitations
	Psychological	Anxiety, Depression
	Social	Social activities, Family influence, Work influence
	Treatment	Treatment compliance, Therapeutic Effect

### The item Pool and the first draft of the scale

Nine core dimensions were identified based on the PRO conceptual model of LDH: anxiety, depression, physical symptoms, functional limitations, social activities, family influence, work influence, therapeutic effect, and treatment compliance. A comprehensive literature review on functional, psychological, and quality of life assessments related to LDH, including both domestic and international studies, was conducted. From this review, a preliminary item pool consisting of 54 items was compiled by integrating elements from existing scales and considering the cultural and lifestyle characteristics specific to the Chinese population.

The item pool was subsequently refined through a validation process involving 20 patients and consultations with six experts from orthopedics, rehabilitation, and psychology. Redundant, ambiguous, and poorly representative items were removed. Overlapping items were merged, specificity was enhanced, 15 non-essential items were deleted, eight items were consolidated to improve clarity, and three new items were added to enrich the content. Through this iterative process, a refined draft scale comprising 34 items was developed.

### Formation of the trial scale

During the development of the LDH trial scale, a comprehensive survey was conducted with 200 patients in the first round. From this cohort, 163 valid responses were collected, resulting in a response rate of 81.5%. To ensure sufficient statistical power, a post hoc power analysis was performed using G-Power software. With an effect size of 0.3 (medium effect) and an alpha error probability of 0.05, the analysis yielded a power of 0.9788, indicating a high probability of detecting a true effect. This analysis supported the adequacy of the chosen sample size, confirming its sufficiency for identifying significant relationships while maintaining data collection efficiency.

The demographic characteristics of the respondents included 71 males and 92 females, with a mean age of 44 ± 12.6 years. Among them, 110 participants (67.5%) held a college degree or higher, and 132 (81.0%) resided in urban areas (Table 2).

**Table 2.** General demographic characteristics of respondents in the first round.

Items	Groups	Number(*n* = 163)	Percentage (%)
Sex	Male	71	43.6
	Female	92	56.4
Age	≤29	23	14.1
	30–39	39	23.9
	40–49	50	30.6
	≥50	51	31.3
Educational background	High school and below	53	32.5
	University	89	54.6
	Graduate student	21	12.9
Place of residence	City	132	81.0
	Towns and villages	31	19.0

Based on data from the initial investigation, five item screening methods were applied to develop the first version of the scale: discrete trend method, correlation coefficient method, critical ratio method, Cronbach's α coefficient method, and factor analysis. Three of these methods were deemed particularly effective for item elimination and for enhancing item independence. Specifically, items exhibiting an absolute correlation coefficient greater than 0.7 were deleted or combined, resulting in the removal of 9 items and retention of 25 items in the final scale (Table 3).

**Table 3.** Screening results of draft items of the scale.

Items	Standard difference	Correlation coefficient	Threshold	Cronbach α coefficient	Factor loading	Retain or delete
1. Do you have back pain or leg pain on weekdays?	0.83	0.38	3.96	0.938	0.79	R
2. How is your lower back pain or leg pain?	0.87	0.53	7.03	0.937	0.54	R
3. At a normal pace, can you walk continuously for more than 15 min?	0.97	0.52	6.35	0.937	0.82	R
4. Do you have difficulty going up stairs because of the herniated lumbar disc?	0.96	0.56	8.01	0.936	0.60	R
5. Because of low back pain, do you have difficulty standing in a normal posture for 20-30 min?	1.07	0.59	6.87	0.936	0.69	R
6. Do you have trouble getting up from the chair by yourself after sitting for a while?	0.81	0.50	6.86	0.937	0.66	R
7. Can you bend forward and pick things up on the ground with your hands?	0.90	0.63	8.52	0.936	0.60	R
8. Do you need to lie down because of your lower back pain?	0.97	0.64	9.51	0.936	0.59	R
9. Do you have trouble getting up in the morning because of your back pain?	0.89	0.56	8.18	0.936	0.78	D
10. Do you have any difficulty turning yourself over in bed?	0.93	0.57	8.21	0.936	0.69	R
11. Does low back pain or leg pain affect your sleep quality?	0.97	0.66	8.79	0.936	0.49	R
12. Does low back pain or leg pain affect your sex life?	1.10	0.68	10.23	0.935	0.61	R
13. Do you have any difficulty getting dressed because of your lower back pain?	0.84	0.68	11.83	0.936	0.78	D
14. Do you have trouble putting on your own socks because of your low back pain?	1.01	0.59	9.53	0.936	0.59	R
15. Do you have trouble doing housework because of your lower back pain?	1.01	0.69	10.67	0.935	0.63	D
16. Does the low back pain or leg pain affect your bowel movement?	0.73	0.50	6.95	0.937	0.55	R
17. Does the low back pain or leg pain make you feel anxious and nervous?	0.99	0.72	10.44	0.935	0.69	R
18. Does low back pain or leg pain make you emotionally unstable?	1.02	0.78	12.75	0.934	0.83	R
19. Are you irritable due to a herniated lumbar disc?	0.95	0.67	10.00	0.935	0.80	D
20. Are you worried because of the herniated lumbar disc?	1.01	0.75	11.17	0.935	0.74	D
21. Does a herniated lumbar disc make you feel lonely and hopeless?	1.09	0.68	10.91	0.935	0.81	R
22. Does a herniated lumbar disc make you listless and depressed?	1.08	0.76	15.22	0.934	0.78	R
23. Do you agree that physical activity can hurt your lower back?	0.92	0.03	0.49	0.941	0.41	D
24. Do you worry that the low back pain will not be cured or will get worse?	1.11	0.58	7.12	0.936	0.55	R
25. Does low back pain or leg pain affect your normal work?	1.20	0.74	12.22	0.935	0.60	R
26. Have your activities (such as parties, travel, shopping, etc.) been affected because of your low back pain?	1.19	0.75	10.57	0.934	0.65	R
27. Does lumbar disc protrusion affect your daily housework?	1.19	0.70	10.50	0.935	0.67	R
28. Can you follow the doctor's orders to visit the hospital regularly for treatment or follow-up visits?	1.00	0.07	0.82	0.941	0.83	D
29. Can you follow the health education of the medical staff to change the unhealthy habits of life?	0.97	0.11	1.31	0.940	0.92	D
30. Can you do rehabilitation exercises at home as required by the doctor?	0.91	0.09	0.68	0.940	0.88	D
31. Are you satisfied with the treatment so far?	1.08	0.41	5.22	0.938	0.80	R
32. Has your quality of life improved compared to before treatment?	0.97	0.49	5.22	0.937	0.55	R
33. Is your pain less than before treatment?	1.23	0.24	2.80	0.940	0.89	R
34. Has your mood improved compared to before treatment?	1.17	0.32	4.14	0.939	0.88	R

Exploratory factor analysis (EFA) was performed on the trial scale data using the principal component method. The Kaiser–Meyer–Olkin (KMO) measure of sampling adequacy was 0.890, exceeding the recommended threshold of 0.6. Additionally, Bartlett's test of sphericity was significant (*p* < 0.001), confirming the suitability of the data for factor analysis.

**Figure 1.**
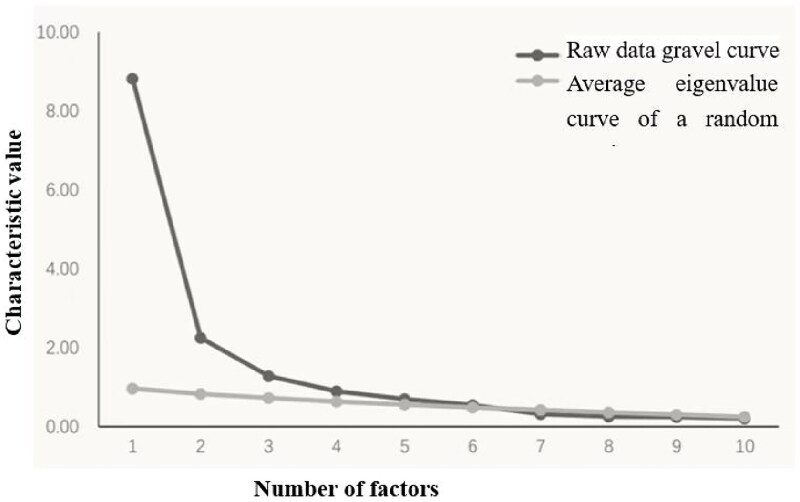
Scale structure preliminary exploration parallel analysis diagram (top 10 factors).

Parallel analysis was employed to determine the optimal number of factors to retain. The scree plot revealed an intersection of the actual data and random eigenvalue curves at the sixth factor (Figure 1), indicating the extraction of six common factors. The variance contribution rates of these factors were 13.39%, 12.48%, 10.95%, 7.67%, 4.46%, and 9.91%, respectively, accounting for a cumulative variance of 58.86% (Table 4).

**Table 4.** Factor analysis results of scale structure were preliminarily explored.

Fields	Dimensions	Items	Variance (%)	Cumulative variance (%)
Social	Factor 1	8,12,25,26,27	13.39	13.39
Psychological	Factor 2	17,18,21,22,24	12.48	25.88
Physiological	Factor 3	6,7,10,11,14,16	10.95	36.82
Therapeutic	Factor 4	31,32,33,34	9.91	46.73
Physiological	Factor 5	3,4,5	7.67	54.40
Physiological	Factor 6	1,2	4.46	58.86

Six experts from orthopedics, rehabilitation, psychology, and related fields reviewed the structure and items of the trial scale. Combined with the EFA results, it was noted that item distribution was unbalanced and that only two items under factor 5 reflected physical symptoms. Consequently, specific revisions were recommended.

Firstly, separate assessment of back pain and leg pain was proposed. To implement this, items such as 'Do you have low back pain on weekdays?', 'How is your back pain?', 'Do you have leg pain on weekdays?', 'How does your leg hurt?', and 'Have you ever experienced numbness in your lower extremities?' were revised or added, totaling five items. Secondly, factors 3 and 4, both reflecting physical dysfunction, were merged into a single dimension. Moreover, factor 1, representing the impact of LDH on work, life, and outdoor activities, was expanded to include travel-related impact by adding the item 'Does low back pain or leg pain affect your traveling or outing?'

Following these modifications, the trial scale was refined to include 29 items covering four fields and nine dimensions consistent with the conceptual model. This revision is believed to enhance the comprehensiveness and accuracy of the scale in assessing the multifaceted impact of LDH on patients' lives, thereby improving its utility and validity.

### Formation of the formal scale

In the second round of the survey, 350 valid questionnaires were collected from a total of 371 patients with LDH, resulting in an effective recovery rate of 94.3%. Among the respondents, 155 were male and 195 were female, with a mean age of 42.5 ± 13.3 years. Of these, 242 participants (69.1%) had attained a college education or higher, and 272 (77.7%) were urban residents (Table 5).

**Table 5.** General demographic characteristics of the respondents in the second round.

Items	Groups	Number (*n* = 350)	Percentage (%)
Sex	Male	155	44.3
	Female	195	55.7
Age	≤29	60	17.1
	30–39	84	24.0
	40–49	89	25.4
	≥50	117	33.4
Educational Background	High school and below	108	30.9
	University	190	54.3
	Graduate student	52	14.8
Place of Residence	City	272	77.7
	Towns and villages	78	22.3

The items of the trial scale were re-screened using the same selection methods and exclusion criteria as in the first round. The screening results indicated that no items required deletion from the scale (Table 6).

**Table 6.** Trial scale item screening results.

Items	Standard difference	Correlation coefficient	Threshold	Cronbach α coefficient	Factor loading	Retain or delete
1. Do you have back pain on weekdays?	1.01	0.48	9.31	0.950	0.63	R
2. How is your back pain?	0.91	0.61	12.79	0.949	0.48	R
3. Do you have leg pain on weekdays?	1.16	0.56	12.28	0.950	0.82	R
4. How does your leg hurt?	1.06	0.58	12.41	0.949	0.72	R
5. Have you ever experienced numbness in your lower extremities?	1.18	0.52	10.00	0.950	0.63	R
6. Do you need to lie down because of your back pain?	1.09	0.64	13.18	0.949	0.45	R
7. Can you bend forward and pick things up on the ground with your hands?	1.07	0.68	14.18	0.948	0.62	R
8. Do you have trouble getting up from the chair by yourself after sitting for a while?	0.84	0.63	13.36	0.949	0.72	R
9. Do you have difficulty turning yourself over in bed because of the pain in your back?	0.79	0.63	11.30	0.949	0.82	R
10. Is it difficult for you to stand in a normal posture for 20–30 min due to back pain?	0.76	0.61	11.79	0.949	0.81	R
11. Do you have trouble putting on your own socks because of your low back pain?	0.84	0.68	12.61	0.948	0.74	R
12. At a normal pace, can you walk continuously for more than 15 min?	0.97	0.70	12.22	0.948	0.44	R
13. Do you have any difficulty going up stairs because of the herniated lumbar disc?	0.91	0.70	13.52	0.948	0.59	R
14. Does low back pain or leg pain affect your sleep quality?	0.97	0.67	12.47	0.948	0.66	R
15. Does low back pain or leg pain affect your bowel movement?	0.67	0.45	7.24	0.950	0.63	R
16. Does low back pain or leg pain affect your sex life?	1.10	0.67	13.53	0.948	0.54	R
17. Does low back pain or leg pain make you emotionally unstable?	1.11	0.74	17.47	0.948	0.82	R
18. Does a herniated lumbar disc make you listless and depressed?	1.10	0.77	17.56	0.947	0.82	R
19. Does a herniated lumbar disc make you feel lonely and hopeless?	1.10	0.72	15.91	0.948	0.80	R
20. Does low back pain or leg pain make you feel anxious and nervous?	1.12	0.74	15.20	0.948	0.84	R
21. Do you worry that the low back pain will not be cured or will get worse?	1.23	0.70	16.01	0.948	0.75	R
22. Does the protrusion of lumbar disc affect your daily housework?	1.14	0.83	21.75	0.947	0.66	R
23. Does low back pain or leg pain affect your normal work?	1.18	0.82	21.08	0.947	0.62	R
24. Does low back pain or leg pain affect your participation in daily social activities (parties, visiting friends and relatives)?	1.14	0.84	20.64	0.946	0.64	R
25. Will low back pain or leg pain affect your travel or outing?	1.22	0.82	22.54	0.947	0.66	R
26. Is your pain less than before treatment?	1.01	0.44	8.16	0.951	0.84	R
27. Has your mood improved compared to before treatment?	1.06	0.44	8.25	0.951	0.86	R
28. Are you satisfied with the treatment so far?	0.92	0.46	7.42	0.950	0.85	R
29. Has your quality of life improved compared to before treatment?	1.06	0.57	9.15	0.949	0.76	R

Exploratory factor analysis (EFA) was conducted on the 29-item scale using data from the second round. The Kaiser–Meyer–Olkin measure of sampling adequacy was 0.94, exceeding the acceptable threshold of 0.6. Bartlett's test of sphericity was significant (*p* < 0.001), confirming the suitability of the data for factor analysis.

**Figure 2.**
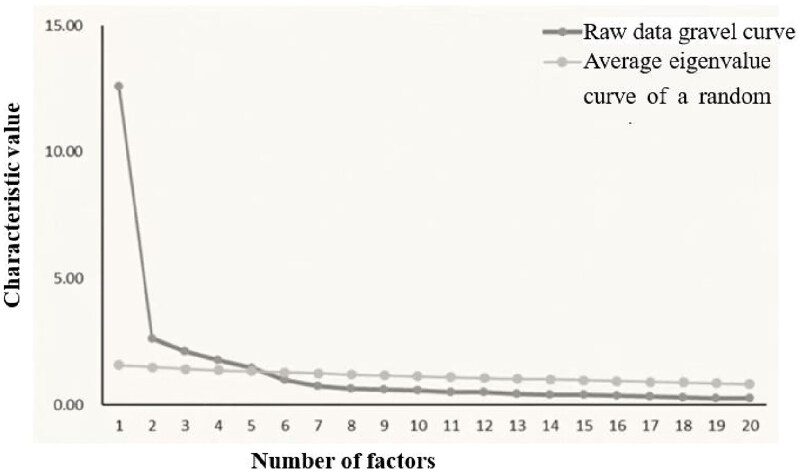
Survey data and random matrix parallel analysis of lithotripsy (first 20 factors).

Parallel analysis was performed to determine the number of factors to extract. The scree plot showed an intersection between the eigenvalue curve of the actual data and that of the randomly generated matrix at the fifth factor (Figure 2), leading to the decision to extract five factors.

The cumulative variance contribution rate of the five factors was 69.07%, indicating that these factors accounted for 69.07% of the total variance. The individual variance contributions were 18.95%, 16.52%, 12.03%, 11.00%, and 10.57%, respectively (Table 7).

**Table 7.** Patients with LDH report outcome formal scale factor rotation factor loading.

Fields	Dimensions	Items	Variance (%)	Cumulative variance (%)
Physical dysfunction	Factor 1	6–16	18.95	18.95
psychological emotion	Factor 2	17–21	16.52	35.47
Social adaptation	Factor 3	22–25	12.03	47.5
Therapeutic effect	Factor 4	26–29	11.00	58.5
Physical symptoms	Factor 5	1–5	10.57	69.07

The factor analysis results revealed a five-dimensional model consistent with the predetermined conceptual dimensions, providing a comprehensive reflection of PRO for LDH. The factors were named according to the items with the highest loadings as follows: (1) Physical dysfunction (11 items), reflecting the impact of LDH on patients' physical function; (2) Psychological emotion (5 items), capturing the influence of LDH on psychological well-being, including anxiety and depression; (3) Social adaptation (4 items), representing the effects on social roles such as work, family, and social interactions; (4) Therapeutic effect (4 items), assessing the extent of treatment improvement and quality of life; and (5) Physical symptom (5 items), describing the frequency and intensity of pain, numbness, and other physical symptoms.

Following two rounds of surveys and item selection, a comprehensive PRO scale for LDH patients was developed, comprising 29 items across five dimensions. The scale is designed for outpatients and inpatients aged over 18 years with a confirmed diagnosis of LDH. A five-level Likert scoring method (values 1–5 corresponding to response options) was employed for each item. Dimension scores were calculated as the average of the respective items, and the total scale score was computed as the average of all items. Higher scores indicated poorer PRO, reflecting a greater impact of the disease on quality of life.

### Verify the reliability and validity of the formal scale

A total of 584 questionnaires were distributed, and 539 valid responses were obtained, resulting in an effective recovery rate of 92.29% (Table 8). The sample comprised 324 males (60.1%) and 215 females (39.9%), with a mean age of 40.2 ± 12.4 years. Among the participants, 398 (73.8%) had attained a university education or higher, 379 (70.3%) resided in urban areas, and 315 (58.4%) reported a monthly income exceeding 6000 yuan. The demographic characteristics of the sample used for test–retest reliability assessment are summarized in Table 9.

**Table 8.** Demographic characteristics of the participants.

	Sort	Number	Percentage (%)
Sex	Male	324	60.1
	Female	215	39.9
Age	≤29	116	21.5
	30–39	160	29.7
	40–49	130	24.1
	≥50	133	24.7
Educational	High school and below	141	26.2
	University (including junior college)	330	61.2
	Graduate student	68	12.6
Residence	City	379	70.3
	Villages	160	29.7
Personal monthly income (¥)	≤3000	98	18.2
	3001–6000	126	23.4
	6001–10,000	156	28.9
	>10,000	159	29.5
Frequency of daily exercise	Everyday	100	18.6
	Frequently	114	21.2
	Occasional	171	31.7
	Little	154	28.6
Working pressure	No	120	22.3
	Little	266	49.4
	Greater	127	23.6
	Great	26	4.8
Smoking	No	351	65.1
	Yes	188	34.9
Diabetes	No	505	93.7
	Yes	34	6.3
Duration of illness	Less than 3 months	113	21.0
	3 months–1 year	132	24.5
	1–3 years	131	24.3
	More than 3 years	163	30.2
Occupational	Mainly manual labor	211	39.1
	Mainly mental work	328	60.9
History of lumbar trauma	Yes	141	26.2
	No	398	73.8
Duration of sitting	Less than 4 h	147	27.3
	4-6 h	162	30.1
	6-8 h	149	27.6
	More than 8 h	81	15.0

**Table 9.** Demographic characteristics of test–retest reliability.

	Total sample (*n* = 539)		Test–retest samples (*n* = 32)		*p*-value
	Number	Percentage (%)	Number	Percentage (%)	
Sex					
Male	324	60.1	21	65.6	0.535
Female	215	39.9	11	34.4	
Age					
≤29	116	21.5	6	18.8	0.934
30–39	160	29.7	10	31.2	
40–49	130	24.1	9	28.1	
≥50	133	24.7	7	21.9	
Education					
High school and below	141	26.2	5	15.6	0.185
University (including junior college)	330	61.2	19	59.4	
Graduate student	68	12.6	8	25.0	
Residence					
City	379	70.3	28	87.5	0.078
Villages	160	29.7	4	12.5	
Personal monthly income (¥)					
≤3000	98	18.2	7	21.9	0.823
3001–6000	126	23.4	6	18.8	
6001–10,000	156	28.9	8	25.0	
>10,000	159	29.5	11	34.3	

### Reliability analysis

The overall Cronbach's α coefficient of the scale was 0.95, with coefficients for individual dimensions all exceeding 0.80. The highest coefficient was observed in the social adaptation dimension (0.94), while the lowest was found in physical symptoms (0.81). The test–retest reliability coefficient for the entire scale was 0.85. Dimension-specific test–retest coefficients ranged from 0.69 for therapeutic effect to 0.87 for physical dysfunction. Except for the therapeutic effect dimension, all reliability coefficients exceeded 0.75, indicating that the scale demonstrated good reliability across most dimensions (Table 10).

**Table 10.** Scale of lumbar disc herniation and reliability coefficients for each dimension.

Dimensions	Cronbach's α	Test–retest reliability
Physical dysfunction	0.91	0.87
Psychological emotion	0.92	0.93
Social adaptation	0.94	0.76
Therapeutic effect	0.89	0.69
Physical symptom	0.81	0.79
Total	0.95	0.85

### Validity analysis

The scale was developed based on a rigorous process incorporating findings from domestic and international studies related to functional, psychological, and quality of life assessments in LDH patients. Content validity was evaluated by a panel of 10 experts specializing in orthopedics, rehabilitation, and psychology. The CVR ranged from 0.6 to 1, and the CVI ranged from 0.9 to 1, indicating strong content validity.

Criterion-related validity was assessed by correlating the new scale with established instruments. The correlation coefficient between the ODI total score and the new scale's total score was 0.74. The correlation between ODI and the physical dysfunction dimension was 0.75. For the SAS, the correlation with the new scale total score was 0.62, and with the psychological emotion dimension was 0.63. All correlations were statistically significant (*p* < 0.01), confirming good criterion-related validity of the formal scale relative to the ODI and SAS (Table 11).

**Table 11.** Results of criterion-related validity analysis.

Correlation coefficient	ODI	SAS
Total	0.74**	0.62**
Physical symptom	0.51**	
Physical dysfunction	0.75**	
Psychological emotion		0.63**
Social adaptation	0.63**	

** Indicates a significant association at the 0.01 level (bilateral).

ODI, Oswestry Disability Index.

SAS, Self-Rating Anxiety Scale.

## Discussion

This study aimed to develop a culturally tailored PRO scale for patients with LDH, capturing their holistic health experience across physical, psychological, and social domains. The final scale, comprising five dimensions and 29 items, was successfully constructed in accordance with the study's original objectives. Specifically, the scale met the goal of establishing a standardized assessment tool that reliably reflects the multidimensional experience of LDH patients. The identified dimensions – physical dysfunction, psychological emotion, social adaptation, Therapeutic Effect, and Physical symptom – corresponded precisely to the critical aspects intended to be addressed, thereby ensuring comprehensive coverage of LDH's impact on patient well-being.

The structural validity of the scale was supported by exploratory factor analysis and parallel analysis, which confirmed its sound factorial structure. These analyses demonstrated that the scale possesses sufficient reliability and validity to detect meaningful variations in disease progression and treatment outcomes. By maintaining sensitivity across diverse dimensions of patient health, the scale fulfilled the initial hypothesis that a reliable, valid, and sensitive tool could be developed to overcome limitations of existing assessment methods.

Beyond its favorable psychometric properties, the developed PRO scale was designed for seamless integration into routine clinical practice. The concise 5-level Likert response format facilitated efficient administration during both outpatient and inpatient visits, allowing healthcare professionals to assess key domains of patient well-being – including physical symptoms, physical dysfunction, psychological emotion, social adaptation, and therapeutic effect – while imposing minimal burden on patients. By providing standardized outcome data, the scale supported clinical decision-making and enabled monitoring of treatment progress, thus assisting in the formulation of individualized intervention strategies. Furthermore, the scale's cultural adaptation—specifically tailored to the unique experiences of Chinese patients – ensured a more contextually relevant and effective assessment approach. Collectively, these features are expected to enhance patient care and quality of life for individuals with LDH, while also establishing a valuable framework for future research and broader clinical application.

### Scale structure and trial scale

The selection of items plays a crucial role in scale development, as it directly influences the quality and effectiveness of the instrument. Robust items are required to demonstrate independence, sensitivity, representativeness, acceptability, and operability. Typically, a combination of expert consultation and statistical screening methods based on classical test theory is employed. In this study, the research team comprised experts from multiple disciplines, including orthopedics, rehabilitation, psychology, and related fields involved in LDH treatment, thereby ensuring comprehensive coverage of relevant patient care aspects.

Following an extensive review of the literature and clinical practices, both domestic and international questionnaires commonly used to assess LDH patients were examined. Furthermore, interviews were conducted with the target population to gain insights into the psychological, physiological, and social impacts of LDH. Based on these sources, an initial item pool was generated by integrating items from two primary origins. The first source included established scales widely used in low back pain assessment, such as the ODI, JOA, RMDQ, SDS, SAS, VAS, McGill Pain Questionnaire, SF-36, EQ-5D, among others, which evaluate dimensions including pain, dysfunction, anxiety, and quality of life. The second source consisted of items developed by the research team informed by patient interviews and the study's objectives. Following expert evaluation and empirical investigation, a preliminary draft containing 35 items was produced.

Subsequently, five screening methods grounded in classical measurement theory were applied to review the preliminary items rigorously. This process led to the construction of a trial scale. Initial exploratory factor analysis combined with parallel analysis extracted six factors that closely corresponded to the original conceptual dimensions. Upon consultation with psychological experts, anxiety and depression factors were merged into a single psychological domain due to their high inter-correlation, collectively representing patients' psychological status. In the social domain, items relating to social activities, family influence, and work influence were consolidated to form a factor reflecting social adaptation. Items related to treatment compliance were deemed non-independent and therefore removed, resulting in a distinct factor representing Therapeutic Effect. Although the distribution of items across factors was somewhat imbalanced at this stage, this was considered acceptable for the preliminary structural exploration characteristic of an initial investigation.

A second round of investigation is planned, utilizing a larger trial scale administered to an expanded sample. This subsequent study will facilitate further item screening and enable more detailed structural analysis of the scale, in accordance with the findings obtained from the initial survey.

### Formal scale

To further refine the items of the trial scale, a second round of investigation was conducted involving a larger cohort of patients diagnosed with LDH. In total, 350 individuals participated in this phase, which exceeded the recommended minimum – ten times the number of scale items – thus ensuring adequate statistical power. To enhance the comprehensiveness and generalizability of the scale, a portion of hospitalized LDH patients was intentionally included. Specifically, 90 inpatients (accounting for 25.7% of the sample) were recruited. The questionnaire required approximately 5 to 10 min to complete, and the effective response rate was 94.3%, indicating that the scale was well understood and acceptable to participants.

Based on the data collected during this second survey, an exploratory factor analysis (EFA) was conducted on the final set of 29 retained items. Parallel analysis revealed that the intersection point between the eigenvalue curve of the actual data and that of the random matrix occurred between the fourth and fifth factors. While extracting only four factors resulted in a cumulative variance contribution rate of 58.49%, this was considered suboptimal. Given that factors with eigenvalues greater than 1 are typically retained in EFA, five factors were ultimately extracted, yielding a cumulative variance contribution rate of 69.07%, which was deemed satisfactory.

The five extracted factors were interpreted as follows:

Factor 1: Physical Dysfunction (10 items) – Reflecting the impact of LDH on patients' physical functioning.

Factor 2: Psychological Emotion (5 items) – Capturing the influence of LDH on psychological states such as anxiety and depression.

Factor 3: Social Adaptation (4 items) – Assessing the impact of LDH on social functioning, including work, family life, and social participation.

Factor 4: Therapeutic Effect (4 items) – Measuring the extent of treatment-related improvement and enhancement in quality of life.

Factor 5: Physical symptom (6 items) – Reflecting the frequency and intensity of pain, numbness, and other physical symptoms associated with LDH.

These results confirmed that the refined formal scale retained a clear and stable five-factor structure, consistent with the multidimensional nature of LDH-related outcomes.

### Verify the reliability and validity of the formal scale

The reliability analysis indicates that the LDH PRO scale possesses strong psychometric properties and is a reliable instrument for assessing patient outcomes. The overall Cronbach's alpha coefficient was 0.95, and all five dimensions demonstrated internal consistency reliability coefficients exceeding 0.80. These values meet and exceed the commonly accepted threshold in clinical and psychological research, where coefficients above 0.80 are generally considered indicative of high reliability. These results are consistent with findings from previous studies on PRO instruments used in chronic pain and musculoskeletal conditions, where similarly high Cronbach's alpha values have been reported, supporting the internal consistency of such scales [31,32].

The scale also demonstrated good test–retest reliability, with coefficients ranging from 0.69 to 0.87. These values suggest that the scale yields stable and consistent results over time, further reinforcing its reliability for repeated clinical use.

In terms of validity, the CVR for individual items ranged from 0.60 to 1.00, while the CVI ranged from 0.90 to 1.00. These values indicate that the items were deemed highly relevant by the expert panel and confirm the scale's strong content validity. These findings are comparable to those of well-established assessment tools for LDH and low back pain, such as the ODI and the RMDQ, which exhibit similarly high content validity metrics [33,34].

Criterion-related validity was also supported by significant correlations between the new scale and existing validated instruments. Specifically, the total score of the LDH PRO scale demonstrated a strong positive correlation with the ODI (*r* = 0.74), and the physical dysfunction dimension correlated significantly with the ODI (*r* = 0.75). Furthermore, the total score correlated with the SAS (*r* = 0.62), while the psychological emotion dimension correlated with the SAS (*r* = 0.63). All correlations were statistically significant (*p* < 0.01), indicating that the new scale effectively captures both physical and psychological aspects of LDH.

These findings support the multidimensional validity of the scale, confirming its ability to comprehensively assess the disease burden experienced by LDH patients. Notably, this aligns with previous studies that emphasize the importance of psychological evaluation in LDH management, highlighting the role of anxiety and depression in exacerbating physical symptoms and impairing patient outcomes [7,8]. The scale's strong alignment with the five-factor structure further affirms its structural validity, providing a robust framework for future clinical and research applications.

### Comparative advantages of the formal scale over existing instruments

Although a wide range of PRO instruments have been developed to evaluate LDH, many conceptualize pain as a singular, overarching symptom. This approach may overlook the complex, multifaceted nature of pain experienced by LDH patients. In recent years, a multi-dimensional assessment of pain – integrating pain-related physical function and psychological components – has been increasingly recommended for the evaluation of chronic pain conditions [35,36]. This is especially pertinent given that existing LDH-specific PRO instruments have generally not been developed in full accordance with the U.S. FDA PRO guidelines [37]. These guidelines emphasize the importance of conducting qualitative research to explore patient experiences and explicitly link those experiences to discrete PRO concepts that are essential for assessing treatment outcomes [38]. In cases of axial low back pain, for example, pain is often diffusely located across multiple regions yet perceived as a singular sensation by patients [39]. Instruments that incorporate patient-derived qualitative data to establish content validity are therefore better positioned to detect meaningful symptom changes in clinical research evaluating therapeutic benefit.

Among currently available instruments, the ODI and the RMDQ are widely used and demonstrate acceptable psychometric performance in assessing physical dysfunction in LDH populations. However, both scales fail to address psychological well-being or treatment effectiveness. The ODI focuses primarily on domains such as mobility, self-care, and daily functional activities, without accounting for mental health status or quality-of-life impacts, as previously noted by O'Brien et al. [18,40]. Likewise, the RMDQ, originally developed by Roland and Morris, centers on functional impairment and lacks constructs that capture emotional or psychological distress – gaps that have been underscored in recent evaluations [19]. As such, although these instruments remain valuable for assessing physical disability, they fall short in capturing the broader psychosocial impacts of LDH.

The JOA score expands slightly on this model by including both subjective and objective symptoms and limitations in daily living. However, similar to the ODI and RMDQ, the JOA score does not encompass psychological health, which has increasingly been recognized as a critical determinant of overall patient well-being and treatment outcomes. Additionally, these traditional instruments are insufficient for assessing treatment efficacy, a dimension essential for long-term patient monitoring and care planning.

Other scales, such as the Numerical Pain Rating Scale (NPRS), ODI, RMDQ, and SF-36, have been assessed for their psychometric properties in LDH populations [16]. While the ODI demonstrates modest advantages in terms of validity and reliability and is more suitable for cross-sectional surveys, the RMDQ is better suited for intervention studies due to its higher responsiveness to clinical changes. The NPRS and JOA may offer some utility in quality-of-life assessments; however, the SF-36, despite its comprehensiveness, imposes significant respondent burden due to its length and complexity. Moreover, recent studies have confirmed the limited responsiveness of the SF-36 in LDH populations [41], further restricting its utility in detecting clinical change over time.

The newly developed formal scale addresses these critical limitations by incorporating two additional dimensions – psychological emotion and Therapeutic Effect – thereby allowing for a more comprehensive evaluation of the LDH patient experience. These enhancements enable clinicians and researchers to assess not only physical impairment, but also emotional burden and therapeutic outcomes, which are essential for capturing the full impact of LDH. Unlike the ODI, RMDQ, and JOA, this scale provides a more integrated framework suitable for both cross-sectional and longitudinal research. It facilitates a nuanced understanding of patient health status, capturing changes across multiple dimensions of well-being.

Consistent with findings in the broader literature, this study reinforces the importance of incorporating assessments of quality of life, mental health, and social functioning into LDH evaluation protocols [42,43]. Compared to more general scales such as the SF-36, the formal scale's streamlined design offers a more efficient yet equally comprehensive assessment framework, reducing burden on both researchers and participants. This balance between brevity and breadth enhances the scale's practicality and applicability in routine clinical practice and research settings, supporting a more holistic and patient-centered approach to LDH management.

## Limitations

Despite the promising results and methodological rigor, this study is subject to several limitations that warrant consideration. First, the sample population was primarily recruited from a large tertiary hospital in an urban setting in China. This recruitment strategy may limit the generalizability of the findings to patients in rural areas, smaller community clinics, or regions with differing healthcare resources and cultural practices. Given the potential influence of sociocultural context on health perception and reporting, future studies should aim to include a more demographically and geographically diverse sample. Incorporating both urban and rural populations across various healthcare tiers through stratified sampling methods could enhance the external validity and broader applicability of the scale.

Second, although the scale demonstrated satisfactory concurrent validity through its correlation with established instruments such as the ODI and SAS, its responsiveness to clinical change—an essential property for longitudinal PRO measures—was not evaluated in this study. Specifically, the ability of the scale to detect changes in patient status before and after therapeutic interventions remains unknown. Future research should adopt longitudinal study designs that assess sensitivity to change over time. This could include repeated measurements at multiple time points, complemented by validated clinical outcome indicators, to determine whether the scale can reliably capture treatment-related improvements or deteriorations in patient condition.

## Conclusion

In summary, the development of this culturally tailored PRO scale for individuals with LDH constitutes a meaningful contribution to the multidimensional assessment of patient health. By integrating physical, psychological, and social domains, the scale offers a comprehensive framework for evaluating the overall disease burden and Therapeutic Effect experienced by LDH patients. Through a combination of qualitative input, expert consensus, and rigorous psychometric analyses – including exploratory factor analysis, reliability testing, and criterion-related validation – the scale has demonstrated strong internal consistency, test–retest reliability, content validity, and structural integrity.

Crucially, the scale fills notable gaps in existing instruments by incorporating dimensions related to psychological well-being and treatment efficacy, which are often overlooked in traditional tools such as the ODI, RMDQ, and JOA. Its concise format, cultural relevance, and clinical practicality further support its utility in routine practice. By facilitating more accurate tracking of patient outcomes and enabling individualized clinical decision-making, the scale aligns with contemporary principles of patient-centered care.

Taken together, this instrument provides an effective, context-specific alternative for the evaluation and management of LDH in Chinese healthcare settings. Future studies aimed at validating the scale's responsiveness to clinical change will further strengthen its applicability in longitudinal research and therapeutic evaluation.

## Data Availability

The data that support the findings of this study are available from the corresponding author upon reasonable request.
